# Longitudinal association of chronic diseases with depressive symptoms in middle-aged and older adults in China: Mediation by functional limitations, social interaction, and life satisfaction

**DOI:** 10.7189/jogh.13.04119

**Published:** 2023-09-29

**Authors:** Derong Huang, Jian Wang, Huiling Fang, Yingjie Fu, Jiaxu Lou

**Affiliations:** 1 Centre for Health Management and Policy Research, School of Public Health, Cheeloo College of Medicine, Shandong University, Jinan, China; 2 NHC KeyLab of Health Economics and Policy Research, Shandong University, Jinan, China

## Abstract

**Background:**

Several previous studies have shown that the development of depression is often accompanied by chronic diseases; although closely related, the mechanism between them is not clear. Here we investigate the potential role of functional limitations, social interaction, and life satisfaction in the relationship between chronic diseases and depressive symptoms in middle-aged and older adults in China.

**Methods:**

We selected 2407 respondents aged ≥45 from the China Health and Retirement Longitudinal Study conducted in 2013, 2015, and 2018. We established panel data to estimate the longitudinal impact of chronic diseases on depressive symptoms and the mediating role of functional limitations, social interaction, and life satisfaction.

**Results:**

Chronic diseases were associated with more depressive symptoms. All of the mediating pathways examined passed functional limitations, and approximately 43.4% of the association between chronic diseases and depressive symptoms was explained by these three mediating variables.

**Conclusions:**

The impact of chronic diseases on depressive symptoms was primarily mediated by functional limitations, and the mediating role of social interaction and life satisfaction was also confirmed. Therefore, attention should be paid to reducing the level of functional limitation in middle-aged and older adults with chronic diseases and improving life satisfaction by increasing social opportunities to alleviate depressive symptoms in middle-aged and older adults.

Ageing is occurring at an unprecedented rate, with an increasing proportion of middle-aged and older adults globally; simultaneously, mental illness is becoming a prominent public health problem for this group [1]. Depression is one of the most common mental disorders among middle-aged and older adults [2]. Research shows that depressive symptoms are related to higher prevalence of chronic physical diseases [3], higher suicide rates [4], and poorer quality of life [1]. Consequently, research has focused on exploring the risk factors and mechanisms of depression. Chronic disease is one of the most important factors affecting depressive symptoms among middle-aged and older adults [5]. Although several studies have confirmed a significant correlation between chronic diseases and depressive symptoms [6,7], few have explored the potential correlations between the two from a longitudinal perspective, especially in the context of China.

As functional limitations are common complications of chronic physical diseases, they have been prioritised in studies on the mechanism interrelating chronic diseases and depressive symptoms. Previous studies have identified a relationship between chronic diseases, functional limitations, and depressive symptoms. The development of chronic diseases can trigger or exacerbate functional limitations [8,9], which is in turn associated with an increase in depression level [10]. A negative impact of functional limitations on depressive symptoms has also been confirmed [11]. However, few studies have addressed the mediating role of chronic disease-related functional limitations and depressive symptoms. Stegmann et al. [12] examined data from a large European cross-sectional survey and determined that functional limitations explain the relationship between physical condition and depressive symptoms. Parajuli et al. [13] first identified the mediating role of functional limitations longitudinally using US data. However, whether functional limitations of middle-aged and older adults in China longitudinally mediate the relationship between chronic diseases and depressive symptoms remains unclear.

Current data suggest that the effect of chronic diseases on depressive symptoms is largely mediated by functional limitations [12,14], but the exact method of this interaction remains unclear. Depression is a mental illness relevant to the field of psychology. Chronic physical diseases and functional limitations refer to physical and physiological function impairments or a pathological state. Physical and mental health exert both direct and indirect impacts. A previous study proposed a conceptual framework that integrates the mechanisms that mediate interactions between physical and mental health [15], emphasising the role of lifestyle choices and social capital and proposed four specific mechanisms, including employment, individual decision-making, lifestyle choices, and social interaction. In view of this framework, we aimed to explore potential pathways ranging from physiological functions to social functions to psychological functions, as well as the roles of social interaction and life satisfaction in the mediating mechanism. Social interaction level is the concentrated embodiment of individual social function and functional limitations are the medium of the correlation between physiological function and social function, while life satisfaction is the buffer affecting the influence of social function on psychological function.

We hypothesised that chronic diseases are longitudinally associated with depressive symptoms and that they affect depressive symptoms through functional limitations, social interaction, and life satisfaction. Our goal was to provide clearer evidence useful for depression prevention and intervention efforts targeting middle-aged and older adults.

## METHODS

### Study design

We conducted a longitudinal study using data from the Chinese Longitudinal Health and Retirement Survey (CHARLS), a longitudinal survey of the Chinese population aged ≥45 years. CHARLS randomly selected a sample of middle-aged and older adults from 450 villages in 28 provinces by using multi-stage probability sampling [16,17]. The baseline survey was conducted in 2011 and completed in 2018. The database covers the basic background, economic status, physical and mental health status, medical service utilisation statistics, and other extensive information regarding middle-aged and older adults in China. We merged three waves of data collected in 2013, 2015, and 2018 into panel data, excluding participants with missing values among the main variables and participants under 45 years of age. We thus included 2407 follow-up subjects, all of whom participated in the three waves.

### Measures

#### Measurement of chronic diseases

Chronic diseases were assessed based on a self-reported item: “Have you ever had a doctor tell you that you have any of the following chronic diseases?” [18]: hypertension, dyslipidaemia, diabetes or high blood glucose, cancer or malignant tumour, chronic lung diseases (such as chronic bronchitis and lung emphysema), liver disease, heart disease (such as coronary heart disease, angina, or congestive heart failure), stroke, kidney disease, stomach disease or other digestive disease, emotional, nervous or psychiatric problems, and memory-related diseases, arthritis or rheumatism, and asthma. The absence of chronic diseases was assigned a value of 0, the presence a value of 1, and the presence of two or more chronic diseases a value of 2, according to the definition of multiple chronic diseases [19].

#### Measurement of functional limitations

Functional limitations were measured using the activities of daily living (ADL) and instrumental ADL (IADL) scales [20,21]. The ADL includes six items: dressing, bathing, dining, getting into/out of bed, toileting, and controlling urination and defecation. IADL comprises five items: household chores, cooking, shopping, financial management, and taking medicine. Scores were assigned according to the answer given for each item (0 = “No, I don't have any difficult”, 1 = “I have difficulty but can still do it”, 2 = “Yes, I have difficulty and need help”, 3 = “I cannot do it”), and we added 11 items to measure the degree of functional limitation. The score could range from 0 to 33, with higher scores representing higher degrees of functional limitation.

#### Measurement of social interaction

Social interaction was assessed from a self-reported item: “Have you done any of these activities in the last month?”. The listed social activities were divided into three levels according to frequency. The higher the score, the lower the frequency. We reversely encoded these measures and determined the level of participation in social activities by summing the frequencies of 10 activities according to the previous studies [22,23]. The score could range from 0 to 30, with higher scores representing higher levels of social interaction.

#### Measurement of life satisfaction

Life satisfaction was assessed from a self-reported item: “How satisfied are you with your life as-a-whole?” [24]. A score of 1-5 was given for evaluation, with higher scores representing lower life satisfaction. We also performed reverse coding (1 = “Not at all satisfied”, 2 = “Not very satisfied”, 3 = “Somewhat satisfied”, 4 = “Very satisfied”, 5 = “Completely satisfied”) so that higher scores represented higher life satisfaction.

#### Measurement of depressive symptoms

Depressive symptoms were measured according to the Center for Epidemiologic Studies Depression (CES-D) Scale [25], which measures depressive symptoms by asking respondents to review their feelings over the past week and rate 10 items related to mood and feelings. Each entry gives four options, ranging from 0 to 3: “Rarely or none of the time (<1 day)”, “Some or a little of the time (1-2 days)”, “Occasionally or a moderate amount of the time (3-4 days)”, or “Most or all of the time (5-7 days)”. We added items 1, 2, 3, 4, 6, 7, 9, and 10 normally and items 5 and 8 in reverse to obtain the level of depressive symptoms. The score could range from 0 to 30, with higher scores representing higher levels of depressive symptoms.

#### Measurement of covariates

We considered age, retirement, residency, marital status, income level, education level, medical insurance, and pensions as potential covariates. We obtained age by subtracting the year of birth from the year of the survey. We then coded retirement status (1 = retired, 0 = not yet retired), residency type (1 = urban, 0 = rural), and marital status (according to cohabitation: 1 = cohabitation with spouse, 0 = no spouse or non-cohabitation with spouse), and categorised educational levels (0 = illiterate, 1 = lower middle school (including not finishing primary school but being able to read and write, or completing sishu/home school, and elementary school), 2 = middle school, and 3 = high school and above).

We obtained income levels by summing wage income and transfer income, adding 1, and then taking the logarithm. Medical insurance was measured by asking “Are you the policy holder/primary beneficiary of any of the types of health insurance listed below?”. Respondents answered whether they participated in any medical insurance program (1 = yes, 0 = no). Pensions were measured by asking “Which pension(s) do you currently receive, expect to receive benefits from, or contribute to?”(1 = yes, 0 = no).

### Analytical strategy

We first examined the normality of the distribution of the main variables according to the symmetry, kurtosis, and the Kolmogorov-Smirnov tests. We then used Pearson correlation analysis to determine the correlations between major variables and fixed-effect panel models to estimate the effect of chronic diseases on depressive symptoms while controlling for individual time-invariant effects and underlying time trends. The fixed-effects model at the individual level focused on the analysis of within-person changes and effectively excluded observed and unobserved interpersonal differences [26]:

Y_it_ = a_0_ + a_1_X_it_ + ∑ _k_a_k_Control_kit_ + μ_i_ + γ_t_ + ε_it_

where Y_it_ represents the depressive symptom level of individual i at time t, X_it_ the chronic disease status of individual i at time t, Control_kit_ a set of control variables, μ_i_ the individual fixed-effect, γ_t_ the year fixed-effect, and ε_it_ the error term. To examine the mediating effect, we developed the following set of procedures, referring to Baron and Kenny's step-up regression method [27]:

M_1it_ = b_0_ + b_1_X_it_ + ∑ _k_b_k_Control_kit_ + μ_i_ + γ_t_ + ε_it_

M_2it_ = c_0_ + c_1_X_it_ + c_2_M_1it_ + ∑ _k_c_k_Control_kit_ + μ_i_ + γ_t_ + ε_it_

M_3it_ = d_0_ + d_1_X_it_ + d_2_M_1it_ + d_3_M_2it_ + ∑ _k_d_k_Control_kit_ + μ_i_ + γ_t_ + ε_it_

Y_it_ = e_0_ + e_1_X_it_ + e_2_M_1it_ + e_3_M_2it_ +  e_4_M_3it_ + ∑ _k_e_k_Control_kit_ + μ_i_ + γ_t_ + ε_it_

**Figure 1:**
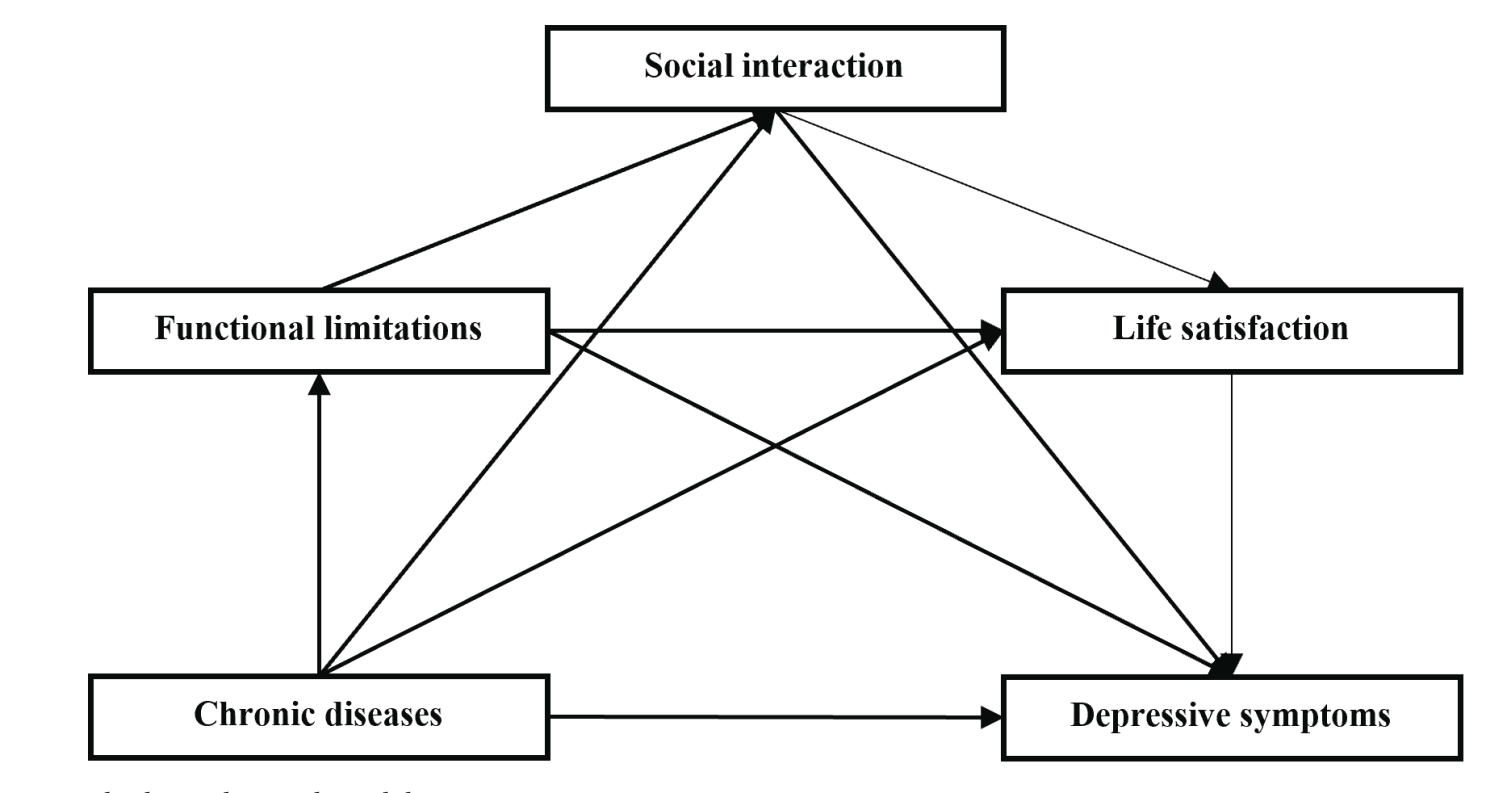
The hypothesised model.

Where M_1it_, M_2it_, and M_3it_ represent the three mediating variables of functional limitations, social interaction, and life satisfaction. This method can effectively calculate the action size of each path. Specifically, the path effect of X passing M_1it_, is only b_1_ × e_2_, the path effect of X passing M_2it_ is only c_2_ × e_3_, the path effect of X passing M_3it_ is only d_3_ × e_4,_ ; then, the path effect of X passing by M_1it_ and M_2it_ is b_1_ × c_2_ × e_3_, the path effect of X passing by M_1it_ and M_3it_ is b_1_ × d_2_ × e_3_, the path effect of X passing by M_2it_ and M_3it_ is c_1_ × d_3_ × e_4_, the path effect of X passing by M_1it_, M_2it_, and M_3it_ is b_1_ × c_2_ × d_3_ × e_3_, and the total effect is e_1_ + b_1_ × e_2_ + c_2_ × e_3_ + d_3_ × e_4_ + b_1_ × c_2_ × e_3_ + b_1_ × d_2_ × e_3_ + c_1_ × d_3_ × e_4_ + b_1_ × c_2_ × d_3_ × e_4_ (**Figure 1**). We then referred to the bootstrap method developed by Preacher and Hayes [28] to test the robustness of the mediation effect and obtained the 95% bias-corrected bootstrap confidence interval (CI) for each significant path after 5000 bootstraps. If the CI does not contain 0, then the indirect effect is valid. We used Stata, version 15.1 (StataCorp LLC, College Station, Texas, USA) for analysis and considered a two-tailed *P* < 0.05 as statistically significant.

## RESULTS

### Descriptive statistics and correlational analyses

Chronic diseases were positively correlated with depressive symptoms (Pearson correlation coefficient (β) = 0.068; *P* < 0.01), functional limitations (β = 0.070; *P* < 0.01), and social interaction (β = 0.030; *P* < 0.05), but were unrelated to life satisfaction (β = -0.005; *P* > 0.1). Additionally, depressive symptoms were positively correlated with functional limitations (β = 0.211; *P* < 0.01) and negatively correlated with social interaction (β = -0.121; *P* < 0.01) and life satisfaction (β = -0.348; *P* < 0.01). There was a significant pairwise correlation between the remaining main outcome variables (**Table 1**).

**Table 1:** Descriptive statistics and correlations among studied variables

Variables	Mean	SD	1	2	3	4	5
1. Depressive symptoms	8.933	6.389	1				
2. Chronic diseases	1.137	0.833	0.068*	1			
3. Functional limitations	1.908	4.125	0.211*	0.070*	1		
4. Social interaction	1.706	2.210	-0.121*	0.030†	-0.138*	1	
5. Life satisfaction	3.235	0.791	-0.348*	0.005	-0.047*	0.037*	1

SD – standard deviation

*P < 0.01.

†*P* < 0.05.

### Fixed-effect regression analyses

**Figure 2:**
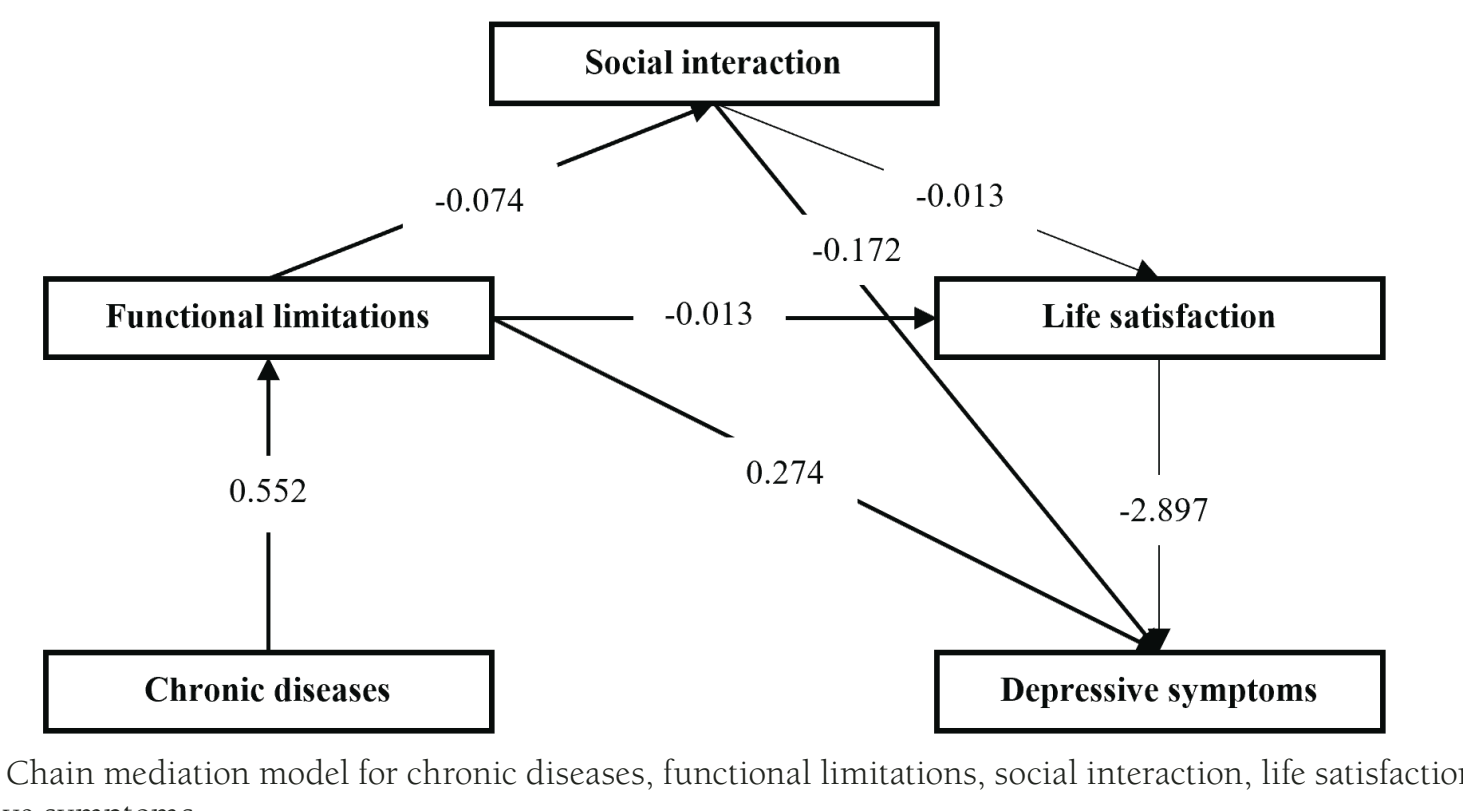
Chain mediation model for chronic diseases, functional limitations, social interaction, life satisfaction, and depressive symptoms.

**Table 2** reports the results of panel fixed-effects models, all of which controlled for both individual time-invariant characteristics and potential time trends. In model 1, chronic diseases positively predicted depressive symptoms, meaning the main effect was significant (β = 0.417; *P* < 0.05). After adding three mediating variables in model 5, chronic diseases no longer significantly predicted depressive symptoms (β = 0.234; *P* > 0.1) and were not associated with social interaction (β = 0.098; *P* > 0.1) or life satisfaction (β = -0.008; *P* > 0.1) and significantly predicted functional limitations only (β = 0.552; *P* < 0.01). Functional limitations negatively predicted social interaction (β = -0.074; *P* < 0.01) and life satisfaction (β = -0.013; *P* < 0.01) and positively predicted depressive symptoms (β = 0.274; *P* < 0.01). Social interaction was a positive predictor of life satisfaction (β = 0.016; *P* < 0.05) and a negative predictor of depressive symptoms (β = -0.172; *P* < 0.01). Life satisfaction was also negatively related to the level of depressive symptoms (β = -2.897; *P* < 0.01). As we included a relatively diverse cohort, models 6 and 7 explored heterogeneity in the middle-aged and older adults, respectively, and showed a significant association between chronic diseases and depressive symptoms in older age groups (β = 0.571; *P* < 0.05), but not in middle age (β = -0.335, *P* > 0.1). We removed non-significant paths to simplify the model and constructed the final multi-mediation model, containing effect values for all paths (**Figure 2**); we found that the path from life satisfaction to depressive symptoms has the largest effect value.

**Table 2:** Results of fixed-effect regression analyses*

	Depressive symptoms	Functional limitations	Social interaction	Life satisfaction	Depressive symptoms		
**Variables**	**Model 1**	**Model 2**	**Model 3**	**Model 4**	**Model 5**	**Model 6**	**Model 7**
Chronic diseases	0.417 (2.36)‡	0.552 (4.45)†	0.098 (1.60)	-0.008 (-0.37)	0.234 (1.48)	-0.335 (-0.98)	0.571 (2.08)‡
Functional limitations			-0.074 (-8.67)†	-0.013 (-3.23)†	0.274 (9.80)†	0.256 (4.51)†	0.269 (5.30)†
Social interaction				0.016 (2.34)‡	-0.172 (-3.72)†	-0.097 (-1.09)	-0.214 (-2.61)†
Life satisfaction					-2.897 (-21.26)†	-2.872 (-10.00)†	-2.800 (-11.90)†
Age	-0.006 (-0.45)	-0.005 (-0.57)	-0.004 (-0.98)	0.000 (0.29)	-0.004 (-0.32)	-0.026 (-0.49)	0.036 (1.33)
Retirement status	-0.338 (-0.66)	0.033 (0.10)	0.350 (1.61)	-0.058 (-0.86)	-0.442 (-0.96)	-2.539 (-2.56)‡	1.250 (1.66)
Log (income)	-0.099 (-2.94)†	-0.025 (-1.13)	0.014 (1.21)	-0.001 (-0.24)	-0.091 (-2.94)†	-0.070 (-1.10)	-0.094 (-1.74)
							
Marital status	-0.251(-0.80)	0.154 (0.84)	0.060 (0.59)	0.047 (1.24)	-0.153 (-0.55)	-0.764 (-1.10)	-0.201 (-0.46)
Pensions	0.004 (0.01)	0.224 (0.89)	0.050 (0.39)	0.027(0.62)	0.020 (0.06)	0.017 (0.03)	0.066 (0.12)
Medical insurance	-0.470 (-0.97)	0.084 (0.27)	0.062 (0.39)	0.045 (0.78)	-0.354 (-0.84)	0.132 (0.16)	-0.061 (-0.08)
Residency	-1.058 (-3.62)†	-0.438 (-2.19)‡	0.207 (1.96)‡	-0.005 (-0.15)	-0.885 (-3.36)†	-1.125 (-2.09)‡	-1.147 (-2.43)‡
Education level	0.008 (0.05)	-0.034 (-0.31)	0.032 (0.63)	-0.017 (-0.90)	-0.023 (-0.17)	-0.026 (-0.09)	-0.131 (-0.51)
Constant	10.350 (9.43)†	0.987 (1.34)	1.710 (4.84)†	2.967 (20.91)†	18.992 (17.84)†	21.361 (6.69)†	15.333 (6.46)†
Individual fixed-effect	Yes	Yes	Yes	Yes	Yes	Yes	Yes
Year fixed-effect	Yes	Yes	Yes	Yes	Yes	Yes	Yes
Number of observations	5330	5330	5330	5330	5330	2460	2870
Number of individuals	2334	2334	2334	2334	2334	1697	1853
Adjusted R^2^	0.015	0.029	0.028	0.039	0.187	0.173	0.194

*Values shown as standardised beta coefficients (standard errors) unless specified differently.

†*P* < 0.01.

‡*P* < 0.05.

### Bootstrap analyses

To verify the robustness of the mediation effect, we further conducted bootstrap analyses with 5000 bootstrapping tests (**Table 3**). Based on the results of the multi-mediation model, we found four statistically significant pathways: functional limitations mediated the relationship between chronic diseases and depressive symptoms (effect value = 0.151; 95% bias correction CI = 0.122-0.224), while chronic diseases affected depressive symptoms through multiple mediators of functional limitations and social interaction (effect value = 0.007; 95% bias corrected CI = 0.006-0.013), through multiple mediators of functional limitations and life satisfaction (effect value = 0.021; 95% bias-corrected CI = 0.004-0.026), and through continuous mediations of functional limitations, social interaction, and life satisfaction (effect value = 0.002; 95% bias-corrected CI = 0.001-0.003). None of the confidence intervals included 0, indicating that all mediation effects were significant. We see that all action pathways of chronic diseases on depressive symptoms were mediated by functional limitations, and the three mediators of functional limitations, social interaction, and life satisfaction can explain 43.4% of the relationship between chronic diseases and depressive symptoms (total indirect effect/main effect).

**Table 3:** Results of bootstrapping analysis*

Effect	Estimate	Boot SE	*P*-value	95% CI for boot
Indirect effect (X → M1 → Y)	0.151	0.026	<0.001	0.122-0.224
Indirect effect (X → M1 → M2 → Y)	0.007	0.002	<0.001	0.006-0.013
Indirect effect (X → M1 → M3 → Y)	0.021	0.005	0.014	0.004-0.026
Indirect effect (X → M1 → M2 → M3 → Y)	0.002	0.001	0.005	0.001-0.003
Total effect	0.181	0.029	<0.001	0.138-0.255

X – chronic diseases, M1 – functional limitations, M2 – social interaction, M3 – life satisfaction, Y – depressive symptoms, SE – standard error, CI – confidence interval

*Number of bootstrap samples for bias-corrected bootstrap confidence intervals: 5000.

## DISCUSSION

We used data from a representative survey of middle-aged and older adults in China to explore the relationship between chronic diseases and depressive symptoms from a longitudinal perspective, revealing key pathways involving functional limitations, social interaction, and life satisfaction and providing new insights into existing literature. We found that chronic diseases have a longitudinal association with depressive symptoms, and that functional limitations, social interaction, and life satisfaction are mediating variables between chronic diseases and depressive symptoms, confirming our initial hypotheses.

We found a significant positive association between chronic diseases and depressive symptoms, consistent with previous findings that having a chronic disease is associated with a higher prevalence of depression [29], as a diagnosis of chronic disease increases the risk of depressive symptoms [5]. However, after adjusting for mediating variables, we found no association between chronic diseases and depressive symptoms, which is not in line with previous studies. For example, a study using data from a three-phase follow-up survey of older adults in China found that chronic diseases had a direct impact on depressive symptoms when cognitive impairment was included in the regression analysis [30]. The discrepancy may be related to differences in sample size and follow-up period. We included a younger cohort, which may compensate chronic diseases better, and therefore, decrease their effect when compared to a sample of older participants. Additionally, we found that chronic diseases lack associations with social interaction and life satisfaction. Notably, although social interaction has no direct association with chronic diseases, it may moderate the relationship between chronic diseases and functional limitations [31].

Our results further showed that the three mediating variables were closely related to and interacted with one another. There is a close relationship between social interaction and functional limitations [32]. An increase in functional limitations will impede physical activities [33] and reduce willingness to actively participate in social activities, which subjects middle-aged and older adults to longer times of self-maintenance and medical rehabilitation at home [34], in turn leading to decreased participation in social activities outside the family [35]. Conversely, previous research showed that increasing the frequency of social interactions helps reduce or prevent functional limitations [36]. Social interaction has a positive impact on life satisfaction [37], because participation in social activities exposes individuals to more social relationships and social resources [38] and a perception of more social support [37], thus improving life satisfaction [39]. Furthermore, our findings suggest that functional limitations negatively affect life satisfaction, possibly due to the negative consequences caused by functional limitations, such as a perceived decline in quality of life, which are favourable indicators of decreased life satisfaction [40].

Furthermore, the results also confirmed that all three mediating variables are directly related to depressive symptoms. Functional limitations are significantly related to depressive symptoms [41], as they can increase the risk of depression [11]. Although a relationship between them has been reported, one study showed that functional limitations have a stronger effect on depressive symptoms than vice versa [42]. Frequency of social engagement is also directly related to depressive symptoms, with one study showing that participating in productive activities can reduce the risk of depression [43]. Life satisfaction is also significantly associated with depressive symptoms [44]. Previous studies have shown that indicators of mental resilience associated with successful aging, such as life satisfaction, mindfulness, and flourishing, are protective factors for depression and can reduce depression levels [45]. We also found a reverse effect of life satisfaction on depressive symptoms.

Finally, and most importantly, we discovered a mediating effect of functional limitations, social interaction, and life satisfaction, with approximately 43.4% of the association between chronic diseases and depressive symptoms explained by these three mediating variables together. Among the variables, functional limitations formed an independent pathway, representing a possible mechanism between illness and depressive symptoms, as suggested by the activity restriction model of depressed affect [46,47]. Moreover, functional limitations explained more of the relationship between chronic diseases and depressive symptoms, consistent with previous findings. One study, for example, suggested that functional limitations explain 17%-64% of the association between chronic physical illness and depressive symptoms [12]. However, the mediating effect of functional limitations can vary depending on the type of chronic physical disease an individual has. For example, the reliability of explanations for functional limitations is strongest for individuals with arthritis and heart disease, which may be related to the painful symptoms of such conditions [48]. Additionally, although the effect values of the three significant pathways were much lower than those of the functional limitations as a single mediator, our results validate the continuous mediating effects of functional limitations, social interaction, and life satisfaction.

Our findings suggest that functional limitations play a very important role in the relationship between chronic diseases and depressive symptoms. All of the mediating pathways examined passed functional limitations. Therefore, chronic diseases affect depressive symptoms mainly through functional limitations. However, we cannot conclude that the relationship between chronic diseases and depressive symptoms is completely mediated through functional limitations, as other mediating variables, such as cognitive impairment [30] and sleep quality [49], also have separate mediating pathways. The continuous mediating mechanism of functional limitations, social interaction, and life satisfaction can be interpreted as follows: chronic diseases increase the risk of functional limitations and may even lead to functional disabilities. Due to physical inconvenience, the frequency of individuals participating in social activities is reduced, and the reduction in frequency of social interaction will then lead to a reduction in individual social relationships and resources, resulting in a reduction in perceived social support that in turn reduces life satisfaction and ultimately aggravates depressive symptoms. Here we examined the mediating effect of individual social functioning as a link between physical and mental health and considered a set of mediating variables collectively to better understand the mechanisms associated with chronic diseases and depressive symptoms and to facilitate development of a coherent set of depression prevention and intervention measures. Based on our findings, we suggest early intervention to prevent the progression of chronic diseases. For middle-aged and older adults with chronic diseases, we should work to maximally reduce the level of functional limitations through exercise rehabilitation technology, nutritional support programs [50], and other approaches. Society or families should take the initiative to provide socialisation opportunities and environments for middle-aged and older adults, especially regarding social activities based on physical activities, such as popularising square dancing, handicrafts, chess and cards, etc. Enriching social relationships not only helps prevent functional limitations [31], but also improves social support, thus promoting increased life satisfaction and ultimately achieving the purpose of reducing depressive symptoms.

This study has some limitations. First, the indicators used here relied primarily on self-reporting by respondents and lacked the support of objective indicators, which could have led to recall bias and other problems. Second, the social activities of older adults vary widely, and evaluating the degree of social participation by simply summing the activities is too one-sided. Further research should distinguish the influence of different types of social participation on the correlation between chronic diseases and depressive symptoms, which would provide a more useful comparison. Third, the quantitative estimate of the severity of chronic diseases was also one-sided. Future research should comprehensively evaluate the severity of chronic diseases to explore the correlation between chronic diseases and depressive symptoms more accurately. Fourth, the chain mediation model was based on parsimonious model, which prevent multiple and bidirectional links, to make the results interpretation easier. Furthermore, there may be some inverse association between the main outcome variables. Finally, we only included a sample of middle-aged and older adults in China, so our results may not be generalisable to other countries or regions. However, our study design is worth replicating in other countries.

## CONCLUSIONS

We determined potential pathway for the longitudinal effects of chronic diseases on depressive symptoms, in which functional limitations mediate a large part of the relationship between chronic diseases and depressive symptoms. We also found a continuous mediating effect of functional limitations, social interaction and life satisfaction, thus improving our understanding of the association between chronic disease and depressive symptoms. Therefore, for middle-aged and older adults suffering from chronic diseases, especially those suffering from multiple diseases, attention should be paid to reducing the level of functional limitation and improving patient life satisfaction by providing more social opportunities and a better social environment, so as to alleviate their depressive symptoms.
